# Association of dietary patterns with diabetes complications among type 2 diabetes patients in Gaza Strip, Palestine: a cross sectional study

**DOI:** 10.1186/s41043-017-0115-z

**Published:** 2017-11-15

**Authors:** Abdel Hamid el Bilbeisi, Saeed Hosseini, Kurosh Djafarian

**Affiliations:** Department of Clinical Nutrition, School of Nutritional Sciences and Dietetics, Tehran University of Medical Sciences, International Campus (TUMS- IC), Tehran, Iran

**Keywords:** Dietary patterns, Factor analysis, Palestine, Type 2 diabetes mellitus

## Abstract

**Background:**

The prevalence of diabetes mellitus is rising worldwide. When diabetes is uncontrolled, it has dire consequences for health and well-being. However, the role of diet in the origin of diabetes complications is not understood well. This study identifies major dietary patterns among type 2 diabetes patients and its association with diabetes complications in Gaza Strip, Palestine.

**Methods:**

This cross sectional study was conducted among 1200 previously diagnosed type 2 diabetes mellitus (both genders, aged 20–64 years), patients receiving care in primary healthcare centers in Gaza Strip, Palestine. Dietary patterns were evaluated using a validated semi-quantitative food frequency questionnaire. Additional information regarding demographic and medical history variables was obtained with an interview-based questionnaire. Statistical analysis was performed using SPSS version 20.

**Results:**

Two major dietary patterns were identified by factor analysis: Asian-like pattern and sweet-soft drinks-snacks pattern. After adjustment for confounding variables, patients in the lowest tertile of the Asian-like pattern characterized by a high intake of whole grains, potatoes, beans, legumes, vegetables, tomatoes and fruit had a lower odds for (High BP, kidney problems, heart problems, extremities problems and neurological problems), (OR 0.710 CI 95% (.506–.997)), (OR 0.834 CI 95% (.700–.994)), (OR 0.730 CI 95% (.596–.895)), (OR 0.763 CI 95% (.667–.871)) and (OR 0.773 CI 95% (.602–.991)) respectively, (*P* value <0.05 for all). No significant association was found between the sweet-soft drinks snacks pattern with diabetes complications.

**Conclusion:**

The Asian-like pattern may be associated with a lower prevalence of diabetes complications among type 2 diabetes patients.

## Background

The prevalence of diabetes mellitus (DM) is steadily increasing everywhere, most markedly in the world’s low and middle-income countries [1]. DM is a metabolic disease characterized by hyperglycemia resulting from defects in insulin secretion, insulin action or both [2]. DM is recognized as an important cause of premature death and disability; it is one of four priority non-communicable diseases targeted by world leaders [3]. Globally, the World Health Organization estimates that, 422 million adults were living with DM in 2014, and projects that DM will be the seventh leading cause of death in 2030 [1]. Most of DM deaths (More than 80%) occur in low and middle-income countries [4]. Due to sophisticated laboratory tests that are usually required to distinguish between type 1 diabetes and type 2 diabetes (T2DM), separate global estimates of diabetes prevalence for type 1 diabetes and T2DM do not exist [1]. In fact, the majority of people with diabetes are affected by T2DM [1]. In Palestine, the prevalence rate of DM was 10.5% in the West Bank and 11.8% in the Gaza Strip among the registered Palestinian refugees [5]. Abu Rmeileh et al. [6] estimated the prevalence of DM in Palestine at 20.8% and 23.4% in 2020 and 2030, respectively. When DM is uncontrolled, it has dire consequences for health and well-being [1]. In addition, diabetes and its complications impact harshly on the finances of individuals and their families and to health systems and national economies through direct medical costs and loss of work and wages [1]. Complications can arise as the disease progresses. Long term complications such as coronary heart disease which can lead to a heart attack, cerebrovascular disease which can lead to stroke, retinopathy (disease of the eye) which can lead to blindness, nephropathy (disease of the kidney) which can lead to kidney failure and the need for dialysis, and neuropathy (disease of the nerves) which increases the chance of foot ulcers, infection and the eventual need for limb amputation may be attenuated by dietary interventions [2]. Dietary patterns is an approach that has been used to investigate diet-disease relations [7–9]. Dietary pattern is potentially useful in making dietary recommendations because overall dietary patterns might be easy for the public to interpret or translate into diets [10]. The etiology of T2DM complications is poorly understood [11]. Diet is one of the lifestyle factors that may play an important role in preventing and managing these conditions [12, 13]. However, few studies have explored the relationship between dietary patterns and diabetes complications. Most studies have examined the associations between individual foods or food groups and nutrients and diabetes complications [14–19], instead of focusing on dietary patterns which is the most sensible approach to test the role of the overall diet on nutrition-related diseases. Therefore, understanding the association between dietary patterns with diabetes complications may be helpful in reducing diabetes-related premature mortality and improve outcomes among T2DM patients. To the best of our knowledge, this is the first study, which examined this association among T2DM patients in Gaza Strip, Palestine. Our study was conducted to identify major dietary patterns among T2DM patients and its association with diabetes complications.

## Methods

### Study participants

This cross sectional study was conducted in the years 2015 and 2016 among a representative sample of Palestinian T2DM patients, selected by a cluster random sampling method. A total of 1200 patients, aged 20–64 years receiving care in the primary healthcare centers (PHCs) in Gaza Strip, Palestine, were included in the study. Gaza Strip is divided into five smaller governorates, which include North Gaza, Gaza City, Mid Zone, Khan Younis and Rafah. The total number of PHCs in Gaza Strip is fifty-four [20]. The PHCs were distributed in each governorate as follows (Eight, fourteen, sixteen, eleven and five PHCs respectively). The study sample was distributed according to the number of PHCs in each governorate as follows (178,311, 356, 244 and 111 patients respectively). Pregnant, lactating women and patients with other types of serious illness such as cancer or acute myocardial infarction were excluded from the study.

### Assessment of anthropometric measurements

Height was measured in all patients (Patients bare footed and head upright) with a measuring rod attached to the balanced beam scale; the height was reported to the nearest 0.5 cm. Weight (kg) was measured using standard scale (Seca); the scale was placed on a hard-floor surface; patients were asked to remove their heavy outer garments and weight was measured and recorded to the nearest 0.1 kg. Furthermore, a stretch-resistant tape was used for measuring waist circumference (WC); WC was measured at the approximate midpoint between the lower margin of the last palpable rib and the top of the iliac crest. The body mass index (BMI) was calculated by dividing weight in kilograms by the square of height in meters.

### Biochemical analysis

After 12 h fasting, venous blood samples were collected from all patients in the PHCs (In the second meeting with the patients) by well-trained and experienced nurses. Venous blood (4.0 ml) was drawn into vacationer tubes and was used for blood chemistry analysis. Serum was separated immediately, and the extracted serum was investigated for (Fasting Plasma Glucose (FPG) mg/dl, High-Density Lipoprotein Cholesterol (HDL-c) mg/dl and Triglycerides (TGs) mg/dl). Mindray BS-300 chemistry analyzer instrument was used for blood chemistry analysis. The laboratory tests were analyzed in a private licensed laboratory.

### Assessment of blood pressure (BP)

BP was measured from the left arm (mmHg) by mercury sphygmomanometer. Three readings on different days, while the patient was seated after relaxing for at least fifteen minutes in a quiet environment, empty bladder. The average of three measurements was recorded.

### Assessment of dietary patterns

Data about dietary patterns were collected by an expert nutritionist, using a validated semi-quantitative food frequency questionnaire (FFQ). The FFQ is relatively easy and inexpensive to administer and can be used to measure dietary intake over a prolonged time period [21, 22]. The FFQ in our study contains a list of 98 food items; it was developed and validated among Palestinian population in 2014 [23]. All participants were asked to estimate the number of times per day, week or month he/she consumed these particular food products and the amount usually eaten per food item by making comparisons with the specified reference portion. Common household measures including measuring cups, spoons and a ruler were shown to assist the participants in the estimation process. The answer categories ranged from 1 to 7 times (7 categories) including never, one to three times per month, one to two times per week, three to four times per week, five to six times per week, one time per day or two to three times per day. Dietary patterns were obtained using factor analysis after the classification of food items into 25 groups (Table 1).

**Table 1:** Food groupings used in the dietary pattern analysis

Food Groups	Food Items
Refined grains	White breads, toasted bread, cooked white rice, pasta (macaroni, spaghetti and the like)
Whole grains	Wheat bread, corn or canned, cooked cereals (as bulgur and the like)
Potatoes	Boiled potatoes
Beans and legumes	Cooked (lentils, chickpeas, black beans or white)
Red meat	(Beef, lamb), other meat (rabbit, duck), cold meats, hamburger
Organ meat	Beef liver or chicken liver, viscera (tripe, brains and the like)
Poultry	Chicken with skin, skinless chicken
Fish and shellfish products	Mixed fried fish, boiled or grilled fish (sardines, tuna), salted fish, canned water fish, canned fish in oil, (oysters, clams, mussels and the like), shellfish (shrimp and the like)
Fast foods	Meats as mortadella, sausage, pizza, pie
Eggs	Eggs
Low-fat dairy product	Skim milk, skimmed milk powder, yogurt
High-fat dairy products	Whole milk, (condensed milk, milk powder), cottage cheese curd or fresh white cheese, cream cheese or portions, ice cream, chocolate powder and the like, chocolate
Vegetables	Cooked spinach, (cabbage, cauliflower, broccoli), lettuce, onions, (carrots, pumpkin), cooked green beans, (eggplant, zucchini, cucumbers), mushrooms, canned vegetables, cooked green peas, garlic, pepper, (parsley, thyme, bay leaves, oregano, cilantro, mint and the like), avocado
Tomatoes	Tomatoes, tomato sauce (ketchup)
Fruit	Lemons, (oranges, grapefruit and the like), bananas, apple or pear, strawberries, (peach, apricot), fresh figs, (watermelon, cantaloupe, pineapple), papaya, grapes, mango, guava, kiwi, dried fruits (as raisins, prunes), fruits in syrup (juices of fruits, peach, pear, pineapple, fig)
Hydrogenated fats	Margarine, butter, mayonnaise
Vegetable oils	Corn oil, sunflower oil
Olive	Olives, olive oil
Nuts and seed products	Nuts (almonds, peanuts, hazelnuts, walnuts and the like), tahini (sesame seeds)
Sugar, sweets, and desserts	Biscuit, (croissant, pastries), shortbread, brownie, (custard, custard pudding), (jams, honey), sugar, tasty type artificial sweeteners
Snacks	Potato chips, bag of chips
Condiments	Spicy (pepper, chili)
Soft drinks	Soft drinks with sugar (as cola, orange, lemon, fanta and the like), low calorie soft drinks, fruit juice packaging
Beverages	Coffee, decaffeinated coffee, tea
Salt and pickles	Salt, pickles

### Assessment of other variables

Additional information regarding demographic, diabetes complications and medical history variables was obtained with an interview-based questionnaire. Diagnosis and classification of diabetes complications was defined according to Palestinian guidelines for diagnosis and management of DM criteria [24]. Past history of diabetes complications and any previous treatment for these complications was recorded by the PHCs doctors on the patients files. In the present study, reports and all relevant documentation, including medical records were checked. Data on physical activity were obtained using the International Physical Activity Questionnaire (IPAQ short version) [25]. The internationally accepted protocol was used to estimate the weekly calorie expenditure expressed as metabolic equivalents per week (MET/wk) or converted to kcal/wk. using the formula kcal = MET × weight ÷ 60. The IPAQ scoring protocol assigns the following MET values to walking, moderate and vigorous intensity activity: 3.3 METs, 4.0 METs, and 8.0 METs, respectively [26]. Pilot study was carried out on thirty patients to enable the researcher to examine the tools of the study. The questionnaire and data collection process were modified according to the result of the pilot study. The data was collected by ten qualified data collectors (Five nurses and five nutritionists) who were given a full explanation and training by the researcher about the study.

### Statistical analysis

Dietary intakes were converted into grams per day. Statistical analysis was performed using SPSS version 20. A major difficulty in studying the relation between dietary patterns and disease outcomes is that dietary patterns cannot be measured directly [27]. One commonly used statistical method for quantifying dietary patterns is factor analysis [28]. Factor analysis was performed to determine the major dietary patterns among T2DM patients. Factor analysis is a useful multivariable statistical tool for investigating dietary patterns [27, 28]. It allows researchers to investigate concepts that are not easily measured directly by collapsing a large number of variables into a few interpretable underlying factors [29, 30]. This data reduction method identifies independent vectors of variables in a correlation matrix and provides scores that allow individuals to be ranked in terms of how closely they conform to the total pattern [31]. In our study, we classified the 98 food items in the FFQ into 25 food groups (Table 1). The food grouping was based on the similarity of nutrient profiles and was somewhat similar to that used in previous studies [32, 33]. A varimax rotation was used to determine the dietary patterns. For defining food groups in each pattern and simplifying dietary pattern tables, factor loads under 0.2 were excluded [34]. The factor load shows the association between food groups and dietary patterns. For determining the number of factors, we considered eigenvalues >1, the scree plot, and the interpretability of the factors. When a food group was loaded in more than one dietary pattern, only the pattern with a higher factor load was considered in the analysis. A factor score for the two major dietary patterns was calculated. This score for each individual shows, the extent to which the dietary pattern is consistent with one of the specified patterns. Higher factor scores show greater consumption of food groups in the pattern and vice versa. The adequacy of data was evaluated based on the value of Kaiser-Meyer-Olkin and Bartlett’s test. The Kaiser-Mayer-Olkin coefficient, which represents the adequacy of the sample size for factor analysis and should be greater than 0.5, was calculated and the obtained value was 0.753 in our study. The obtained dietary patterns scores are expressed as tertiles. The chi-square test was used to determine the significant differences between different categorical variable. The differences between mean were tested by independent samples t-test and one-way ANOVA. Finally, the odds ratio (OR) and confidence interval (CI) for the diabetes complications across tertiles categories of dietary pattern scores were tested by binary logistic regression. *P* value less than 0.05 was considered as statistically significant.

## Results

A total of 1200 patients with T2DM aged 20 to 64 years old (59.8% females, 40.2% males) were included in this study. The characteristics of the study population by sex is shown in Table 2. The results revealed that the mean age (years) for male patients was 49.4 ± 10.6 vs. 49.9 ± 11.3 for females. In addition, for the following factors (Marital status, educational level, family size, monthly income, diabetes duration, type of diabetes medications used, history of smoking, physical activity level (Total MET), BMI, WC, FPG level, TGs level and HDL-c level), the difference was statistically significant in both sexes (*P* value <0.05). With respect to diabetes complications, Table 3 shows that 64.25% of the patients had high BP (≥130/85 mmHg) or treatment of previously diagnosed hypertension, 57.8% of the patients had eyes problems, 10.8% had kidney problems, 7.25% had heart problems, 22.0% had extremities problems and 92.1% of the patients had neurological problems. Furthermore, for the following factors (High BP or treatment of previously diagnosed hypertension, eyes problems, kidney problems and heart problems), the difference was statistically significant in both sexes (*P* value <0.05). Then, we entered food consumption data for the 25 food groups (Table 1) into the SPSS for factor analysis. The scree plot of eigenvalues indicated two major patterns: 1) Asian- like pattern characterized by a high intake of whole grains, potatoes, beans, legumes, vegetables, tomatoes and fruit as well as a low intake of refined grains, sugar, sweets and desserts; 2) Sweet-soft drinks-snacks pattern, characterized by a high intake of refined grains, sugar, sweets, desserts, snacks and soft drinks. The factor loading matrixes for the two major patterns are shown in Table 4. These two major dietary patterns explained 68.3% and 18.1% of the total variance, respectively. In the present study, the dietary patterns scores were classified as tertiles. Then, the characteristics of the study population were evaluated within the tertiles. Table 5 shows that patients in the lowest tertile (T1) of the Asian-like pattern were younger (46.1 ± 11 vs. 53.0 ± 10 years, *P* value <0.001), had received diabetes care instruction more commonly (40.0 vs. 29.5%, P value <0.001), were more frequently physically active (1679 ± 1560 vs. 1228 ± 1506 Total MET, *P* value <0.05), had a lower BMI (28.6 ± 5.6 vs. 31.5 ± 6 kg/m^2^, P value <0.05) and had a lower WC (99.2 ± 16 vs. 108.5 ± 16 cm, P value <0.001) compared to those in the highest tertile (T3). In addition, they had better glucose control and lipid profile. Moreover, the distribution of patients with regard to marital status, educational level, family size, monthly income, diabetes duration and HDL-c level was significantly different across the tertiles of the Asian-like pattern (*P* value <0.05 for all). On the other hand, only the distribution of patients with regard to educational level, family size, type of diabetes medications used, history of smoking and FPG level (mg/dl) was significantly different across the tertiles of the sweet-soft drinks-snacks pattern (P value <0.05). Finally, we computed the OR and CI for the diabetes complications across tertiles categories of dietary patterns scores (Table 6). Our findings demonstrate that after adjustment for confounding variables, patients in the lowest tertile (T1) of the Asian-like pattern characterized by a high intake of whole grains, potatoes, beans, legumes, vegetables, tomatoes and fruit had a lower odds for (High BP, kidney problems, heart problems, extremities problems and neurological problems), (OR 0.710 CI 95% (.506–.997)), (OR 0.834 CI 95% (.700–.994)), (OR 0.730 CI 95% (.596–.895)), (OR 0.763 CI 95% (.667–.871)) and (OR 0.773 CI 95% (.602–.991)) respectively, (*P* value <0.05 for all). No significant association was found between the sweet-soft drinks snacks pattern with diabetes complications.

**Table 2:** Characteristics of the study population by sex

Variables		T2DM (*n* = 1200)	Male (*n* = 482)	Female (*n* = 718)	*P* Value
		No. (%)	No. (%)	No. (%)	
Age (years)	Mean ± SD	49.74 ± 11	49.4 ± 10.6	49.9 ± 11.3	0.238
Marital status	Married	1160 (96.6)	472 (40.7)	688 (59.3)	0.031
	Unmarried	40 (3.3)	10 (25.0)	30 (75.0)	
Educational level	Low education	535 (44.5)	175 (32.7)	360 (67.3)	0.001
	High education	665 (55.4)	307 (46.2)	358 (53.8)	
Family size	Less than five	429 (51.4)	160 (37.3)	269 (62.7)	0.073
	Five or more	771 (64.2)	322 (41.8)	449 (58.2)	
Monthly income	≤ 2000 (NIS)	1054 (87.8)	392 (37.2)	662 (62.8)	0.001
	> 2000 (NIS)	146 (12.1)	90 (61.6)	56 (38.4)	
Diabetes duration (years)	Less than five	316 (26.3)	131 (27.2)	185 (25.8)	0.004
	Five to ten	434 (36.1)	196 (40.7)	238 (33.1)	
	More than ten	450 (37.5)	155 (32.1)	295 (41.1)	
Use diabetes medications	Yes	1200 (100)	482 (40.2)	718 (59.8)	–
Type of diabetes medications used	Diabetes pills	491 (40.9)	215 (43.8)	276 (56.2)	0.020
	Insulin injections	628 (52.3)	244 (38.9)	384 (61.1)	
	Pills & injections	81 (6.75)	23 (28.4)	58 (71.6)	
Received diabetes care instructions	Yes	574 (47.8)	237 (41.3)	337 (58.7)	0.242
	No	626 (52.1)	245 (39.1)	381 (60.9)	
History of smoking	Yes	162 (13.5)	160 (98.8)	2 (1.2)	0.001
	No	1038 (86.5)	322 (31.0)	716 (69.0)	
History of alcohol intake	No	1200 (100)	482 (40.2)	718 (59.8)	–
Physical activity (Total MET)	Mean ± SD	1430.1 ± 1519	1753.2 ± 1663	1213.2 ± 1373	0.001
Body mass index (kg/m^2^)	Mean ± SD	30.22 ± 6.2	28.32 ± 5.1	31.5 ± 6.6	0.001
Waist circumference (cm)	Mean ± SD	104.7 ± 16.9	101.2 ± 14.6	107.1 ± 18	0.001
Fasting plasma glucose (mg/dl)	Mean ± SD	169.6 ± 29.1	168.5 ± 27.5	170.3 ± 30.1	0.046
Triglycerides level (mg/dl)	Mean ± SD	160.4 ± 52.9	154.2 ± 50.0	164.6 ± 54.4	0.006
HDL-cholesterol level (mg/dl)	Mean ± SD	45.1 ± 8.2	42.4 ± 6.3	46.9 ± 8.9	0.001

Data are expressed as means ± SD for continuous variables and as percentage for categorical variables. The differences between means were tested by using independent sample t test. The chi-square test was used to examine differences in the prevalence of different categorical variable. P value less than 0.05 was considered as statistically significant. SD, stander deviation

**Table 3:** Distribution of diabetes complications for the study population by sex

Variables		T2DM (*n* = 1200)	Male (*n *= 482)	Female (*n* = 718)	*P* Value
		No. (%)	No. (%)	No. (%)	
High BP (≥130/85 mmHg) or treatment of previously diagnosed hypertension	Yes	771 (64.25)	289 (37.4)	482 (62.6)	0.001
	No	429 (35.75)	193 (45.0)	236 (55.0)	
Eyes problems	Yes	694 (57.8)	228 (32.9)	466 (67.1)	0.001
	No	506 (42.1)	254 (50.2)	252 (49.8)	
Kidney problems	Yes	130 (10.8)	34 (26.2)	96 (73.8)	0.001
	No	1070 (89.1)	448 (41.9)	622 (58.1)	
Heart problems	Yes	87 (7.25)	23 (26.4)	64 (73.6)	0.004
	No	1113 (92.7)	459 (41.2)	654 (58.8)	
Extremities problems	Yes	264 (22.0)	109 (41.3)	155 (58.7)	0.362
	No	936 (78.0)	373 (39.9)	563 (60.1)	
Neurological problems	Yes	1106 (92.1)	439 (39.7)	667 (60.3)	0.149
	No	94 (7.83)	43 (45.7)	51 (54.3)	

Data are expressed as percentage for categorical variables. The chi-square test was used to examine differences in the prevalence of different categorical variable. P value less than 0.05 was considered as statistically significant

**Table 4:** Factor loading matrix for major dietary patterns

Food Groups	Dietary patterns	
	Asian-like pattern	Sweet-soft drinks-snacks pattern
Refined grains	0.245	0.271
Whole grains	0.206	–
Potatoes	0.208	–
Beans and legumes	0.223	–
Red meat	–	–
Organ meat	–	–
Poultry	–	–
Fish and shellfish products	–	–
Fast foods	–	–
Eggs	–	–
Low-fat dairy product	–	–
High-fat dairy products	–	–
Vegetables	0.323	–
Tomatoes	0.229	–
Fruit	0.985	–
Hydrogenated fats	–	–
Vegetable oils	–	–
Olive	–	–
Nuts and seed products	–	–
Sugar, sweets, and desserts	0.209	0.249
Snacks	–	0.228
Condiments	–	–
Soft drinks	–	0.998
Beverages	–	–
Salt and pickles	–	–
Variance explained (%)	68.302	18.183

Values less than 0.2 were omitted for simplicity. Total variance explained by two factors: 86.485

**Table 5:** Characteristics and dietary intakes of study population by Tertile (T) categories of dietary pattern scores

Variables	Asian-like pattern			*P* Value	Sweet-soft drinks-snacks pattern			*P* Value
	T1	T2	T3		T1	T2	T3	
Age (years)								
Mean ± SD	46.1 ± 11	50.0 ± 10	53.0 ± 10	0.001	49.5 ± 11	48.8 ± 11	50.8 ± 10	0.518
Gender %								
Males	34.6	31.1	34.3	0.721	30.3	33.6	36.0	0.057
Females	32.5	34.8	32.7		35.3	33.2	31.5	
Marital status %								
Married	32.9	33.8	33.3	0.001	33.5	33.1	33.4	0.927
Unmarried	45.5	20.0	35.0		27.5	40.0	32.5	
Educational level %								
Low education	26.5	32.9	40.6	0.001	31.0	31.2	37.8	0.023
High education	38.8	33.7	27.5		35.2	35.0	29.8	
Family size %								
Less than five	31.4	30.8	37.8	0.004	37.6	33.3	29.1	0.001
Five or more	34.4	34.8	30.8		31.0	33.3	35.7	
Monthly income (NIS) %								
≤ 2000 (NIS)	32.5	33.1	34.4	0.047	33.0	32.9	34.1	0.362
> 2000 (NIS)	39.1	34.9	26.0		35.6	36.3	28.1	
Diabetes duration (years) %								
Less than five	39.6	30.0	30.4	0.001	38.3	29.7	32.0	0.138
Five to ten	36.6	35.4	28.0		32.3	38.0	29.7	
More than ten	25.8	33.5	40.7		30.9	31.3	37.8	
Type of diabetes medications used %								
Diabetes pills	34.2	34.0	31.8	0.163	37.0	34.8	28.2	0.014
Insulin injections	33.8	33.9	32.3		31.0	32.0	37.0	
Pills and injections	24.7	24.7	50.6		28.4	34.6	37.0	
Received diabetes care instructions %								
Yes	40.0	30.5	29.5	0.001	34.0	33.3	32.7	0.099
History of smoking %								
Yes	38.9	29.6	31.5	0.983	25.3	29.7	45.0	0.001
Physical activity (Total MET)								
Mean ± SD	1679 ± 1560	1382 ± 1458	1228 ± 1506	0.006	1435 ± 1397	1528 ± 1633	1325 ± 1514	0.229
Body Mass Index (kg/m^2^)								
Mean ± SD	28.6 ± 5.6	30.4 ± 5	31.5 ± 6	0.005	29.4 ± 6	30.0 ± 6	31.2 ± 6	0.347
Waist circumference (cm)								
Mean ± SD	99.2 ± 16	106.5 ± 16	108.5 ± 16	0.001	102.7 ± 17	104.3 ± 17	107.2 ± 16	0.146
Fasting plasma glucose (mg/dl)								
Mean ± SD	168.2 ± 26	169.6 ± 28	171.0 ± 31	0.044	168.4 ± 28	168.1 ± 27	172.3 ± 30	0.001
Triglycerides level (mg/dl)								
Mean ± SD	156.6 ± 53	158.0 ± 54	166.7 ± 50	0.645	148.8 ± 48	160.2 ± 54	172.3 ± 52	0.051
HDL-cholesterol level (mg/dl)								
Mean ± SD	46.6 ± 8	45.4 ± 8.4	43.3 ± 8	0.005	46.0 ± 8.5	45.6 ± 8.1	43.6 ± 8	0.092

ANOVA test was used for quantitative variables and chi-square for qualitative variables. P value less than 0.05 was considered as statistically significant. SD, stander deviation

**Table 6:** Odd ratio and confidence interval for the diabetes complications across tertiles categories of dietary pattern scores

Asian-like pattern					Sweet-soft drinks-snacks pattern				
T1	T2	T3	*P* value	OR (95%CI)	T1	T2	T3	*P* value	OR (95%CI)
High BP (≥130/85 mmHg) or treatment of previously diagnosed HTN (64.25%)									
30.7	31.9	37.4	0.001	0.773 (.679–.879)	26.6	35.9	37.5	0.001	0.648 (.569–.739)
Adjusted*			0.048	0.710 (.506–.997)	Adjusted*			0.561	1.061 (.868–1.298)
Eyes problems (57.8%)									
33.4	32.9	33.7	0.162	0.920 (.819–1.034)	32.3	32.6	35.1	0.375	0.949 (.846–1.065)
Adjusted*			0.542	0.959 (.840–1.096)	Adjusted*			0.050	1.148 (1.000–1.317)
Kidney problems (10.8%)									
27.6	36.2	36.2	0.604	1.054 (.863–1.288)	35.4	23.9	40.7	0.025	0.828 (.702–977)
Adjusted*			0.042	0.834 (.700–.994)	Adjusted*			0.084	0.853 (.711–1.022)
Heart problems (7.25%)									
25.3	25.3	49.4	0.186	0.858 (.685–1.076)	35.6	23.0	41.4	0.123	0.855 (.701–1.043)
Adjusted*			0.002	0.730 (.596–.895)	Adjusted*			0.298	0.894 (.723–1.104)
Extremities problems (22.0%)									
28.0	32.0	40.0	0.131	0.887 (.758–1.037)	29.9	35.2	34.9	0.355	0.939 (.822–1.073)
Adjusted*			0.001	0.763 (.667–.871)	Adjusted*			0.616	1.039 (.893–1.209)
Neurological problems (92.1%)									
33.8	33.4	32.8	0.155	0.823 (.630–1.076)	32.9	33.2	33.9	0.003	1.443 (1.132–1.840)
Adjusted*			0.042	0.773 (.602–.991)	Adjusted*			0.182	1.149 (.937–1.407)

The OR and CI for the diabetes complications across tertiles categories of dietary pattern scores were tested by binary logistic regression. *Adjusted for age, gender, marital status, educational level, family size, monthly income, diabetes duration, type of diabetes medications used, received diabetes care instructions, history of smoking, physical activity (Total MET), BMI (kg/m^2^), WC (cm), FPG level (mg/dl), TGs level (mg/dl) and HDL-c level (mg/dl). P value less than 0.05 was considered as statistically significant. OR, odds ratio; CI, confidence interval

## Discussion

To the best of our knowledge, this is the first study which describes the dietary patterns among T2DM patients and its association with diabetes complications in Gaza Strip, Palestine. With the use of dietary data from the FFQ, two major dietary patterns were identified in the present study by factor analysis. 1) Asian-like pattern characterized by a high intake of whole grains, potatoes, beans, legumes, vegetables, tomatoes and fruit as well as a low intake of refined grains, sugar, sweets and desserts; 2) Sweet-soft drinks-snacks pattern characterized by a high intake of refined grains, sugar, sweets, desserts, snacks and soft drinks. The main findings of this study indicate that, after adjustment for confounding variables, the Asian-like pattern may be associated with a lower prevalence of diabetes complications (High BP, kidney problems, heart problems, extremities problems and neurological problems) among T2DM patients in Gaza Strip, Palestine. The prevalence of DM is rising worldwide [1]. In fact, very few studies have explored the relationship between dietary patterns and diabetes complications in patients with T2DM, which made the comparison of our results with previous studies difficult. Most studies have examined the associations between individual foods or food groups and nutrients and diabetes complications [14–19] instead of focusing on dietary patterns, which is the most sensible approach to test the role of the overall diet on nutrition-related diseases.

Our findings demonstrate that 64.25% of the patients had high BP (≥130/85 mmHg) or treatment of previously diagnosed hypertension. Hypertension is a major risk factor for developing cardiovascular disease, stroke, and kidney disease [35]. The Asian-like pattern in our study may be associated with a lower prevalence of hypertension. Ndanuko et al. [36] in a meta-analysis identified three dietary patterns: Healthy dietary patterns such as the Dietary Approaches to Stop Hypertension (DASH) diet, Nordic diet and Mediterranean diet. These dietary patterns are rich in fruit, vegetables, whole grains, legumes, seeds, nuts, fish and dairy. The author concluded that these dietary patterns were inversely associated with high BP. The results of our study support these findings. The DASH dietary pattern, is rich in fruits and vegetables, low fat dairy products, whole grains, fish, poultry, beans, seeds and nuts. It is low in sodium, added sugars, sweets, fats and red meats. The DASH dietary pattern is a recognized treatment for hypertension, stroke and heart disease [37].

Our findings show that 10.8% of the patients had kidney problems. In addition, we found a significant inverse association between the Asian-like pattern with kidney problems. Khatri et al. [38] examined the associations of varying levels of adherence to the Mediterranean diet on long-term kidney function in an observational, community-based prospective study and reported that adhering to the diet may significantly reduce the risk of developing chronic kidney disease. The Mediterranean dietary pattern is characterized by a high intake of vegetables, legumes, fruits, nuts, cereals and olive oil; a moderately high intake of fish; a low-to-moderate intake of dairy products; a low intake of saturated fats, meat and poultry; and a regular but moderate intake of wine during meals. Lin et al. [39] has evaluated the relationship between the DASH dietary pattern and nephropathy in diabetic patients. The author found that adherence to a DASH dietary pattern was associated with lower odds of estimated glomerular filtration rate decline compared with those without a DASH dietary pattern. The results of our study support these findings.

Our data revealed that 57.8% of the patients had eyes problems. Furthermore, no significant association was found between the dietary patterns with eyes problems. Díaz-López et al. [11] in a clinical trial have evaluated the role of dietary patterns on the incidence of microvascular diabetes complications in an elderly Mediterranean population with T2DM. The author hypothesized that a nutritional intervention based on the Mediterranean diet would have greater protective effect on diabetic retinopathy and nephropathy than a low-fat control diet. After 6.0 years of median follow-up, the author concluded that a Mediterranean diet enriched with extra-virgin olive oil may protect against diabetic retinopathy but not diabetic nephropathy. Our study not adjusted for other confounding variables such as genetics factors, and different diagnostic methods and criteria used, which could contribute to these results.

Moreover, our findings demonstrate that 7.25% of the patients had heart problems. In our study, the Asian-like pattern may be associated with a lower prevalence of heart disease. Aljefree et al. [40] in a systematic review reported three dietary patterns: Western dietary pattern, Mediterranean and DASH dietary patterns. The findings of this study demonstrate a positive association of the Western dietary pattern with the increased risk of coronary heart disease and strokes, among adults in the Middle East and North Africa region. Conversely, the author concluded that adherence to Mediterranean or DASH dietary patterns or their individual food components was associated with a decreased risk of coronary heart disease and it is associated risk factors [40]. The results of our study support these findings. On the other hand, the results of our study revealed that the Asian-like pattern may be associated with a lower prevalence of extremities and neurological problems. Neuropathy it is a disease of the nerves, which increases the chance of foot ulcers and the eventual need for limb amputation [2]. The diabetic neuropathies are among the most common complications of diabetes [2]. The epidemiology of diabetic neuropathy is unclear because of inconsistent definitions of what constitutes neuropathy; frequencies of 10–100% of patients have been reported [24]. In our study, 22.0% of the patients had extremities problems and 92.1% had neurological problems. Increasing diabetes duration and patients’ age, hypertension, obesity, dyslipidemia and smoking could contribute to these results. Nutrition is a critical component of the healing of diabetic foot ulcers, particularly as it relates to immune function, malnutrition, glycemic control, and weight loss and weight maintenance. Little et al. [41] show that, nutrition assessment and intervention can help patients with diabetic foot ulcers and maximize their nutritional status to promote wound healing. The previous dietary patterns are different from those obtained in our study. This can be explained by demographic, cultural and ethnic differences. In our study, the inverse association between Asian-like pattern with diabetes complications could be attributed to pattern’s healthy ingredients including vitamins, dietary fibers, potassium, magnesium and antioxidants. These nutrients have been independently associated with reduced risks of diabetes complications. Magnesium, potassium, and dietary fibers in many fruits and vegetables may be associated with a lower risk of hypertension, reduce cholesterol concentrations and other cardiovascular risk factors in patients with T2DM [42]. In addition, anti-inflammatory and antioxidant effects in these foods may have beneficial effects in alleviating inflammation and oxidative stress, and decreasing insulin resistance and secretion, which are pathogenic factors in diabetes [43] and diabetes complications [44]. Furthermore, vegetables, legumes, and fruits contain minerals, polyphenols, and other phytochemicals that combat oxidative stress, inflammation and insulin resistance [45, 46]. In our study, the Asian-like pattern has been shown to be the healthiest dietary pattern and is quite close to that diet, which generally recommended as a healthy dietary pattern with low in animal foods, saturated fat, trans fat, cholesterol and simple sugar, which may be associated with a higher risks of diabetes and its complications [47]. Actually, the relationship between dietary patterns with diabetes complications need more studies in the future. The main limitations of this study is its cross sectional design; the causal relationship could not be determined, and it limits the generalizability of our results. In addition, the possibility of recall bias and misreporting by using FFQ assessment of dietary patterns are other limitations. Furthermore, unfortunately we do not have measures of total cholesterol, low-density lipoprotein cholesterol and glycated hemoglobin as a marker of diabetes control. The main strength of our study was its being the first study, which shows the dietary patterns among T2DM patients and its association with diabetes complications in Gaza Strip, Palestine, and its large sample size.

## Conclusions

We conclude that, the Asian-like pattern may be associated with a lower prevalence of diabetes complications among T2DM patients in Gaza Strip, Palestine. Further future studies are required to confirm these findings.

## Abbreviations

BMI: Body mass index
BP: Blood pressure
CI: Confidence interval
DASH: Dietary approaches to stop hypertension
DM: Diabetes mellitus
FFQ: Food frequency questionnaire
FPG: Fasting plasma glucose
HDL-c: High density lipoprotein cholesterol
IPAQ: International physical activity questionnaire
MET/wk.: metabolic equivalents per week
OR: Odds ratio
PHCs: Primary health care centers
SPSS: Statistical package for the social sciences
T: Tertile
T2DM: Type 2 diabetes mellitus
TGs: Triglycerides
WC: Waist circumference
